# A Randomized, Double-Blind, Placebo-Controlled Clinical Trial of a Mouthwash Containing *Glycyrrhiza uralensis* Extract for Preventing Dental Caries

**DOI:** 10.3390/ijerph19010242

**Published:** 2021-12-26

**Authors:** Yu-Rin Kim, Seoul-Hee Nam

**Affiliations:** 1 Department of Dental Hygiene, Silla University, Busan 46958, Korea; dbfls1712@hanmail.net; 2 Department of Dental Hygiene, College of Health Sciences, Kangwon National University, Samcheok 25949, Korea

**Keywords:** preventive effect, dental caries, *Glycyrrhiza uralensis* extract, mouthwash, randomized controlled trial

## Abstract

This study sought to confirm the effect of using a mouthwash containing *Glycyrrhiza uralensis* extract for oral health management by investigating changes in the pH of dental plaque and bacteria that cause dental caries. A randomized, double-blind, placebo-controlled study was conducted on 60 subjects categorized in either the *Glycyrrhiza uralensis* extract gargle group (*n* = 30) or the saline gargle group (*n* = 30). Scaling was conducted in order to ensure the homogeneity of the oral environment, while gargling was performed once daily before the subjects went to bed for 5 days based on the group. Caries activity was assessed using the Cariview test, while detection of the bacteria that cause dental caries was confirmed using microbiological analysis. All clinical measurements and evaluations were conducted by two trained dental hygienists under the supervision of a dentist. Based on the analysis of dental caries activity and dental caries-causing bacteria, the *Glycyrrhiza uralensis* extract gargle group showed a clear decrease in bacteria compared to the saline gargle group. *Glycyrrhiza uralensis* extract demonstrated no risk of tooth demineralization. It also showed excellent antibacterial activity through inhibition and effective reduction of bacteria that cause dental caries. Therefore, the mouthwash containing *Glycyrrhiza uralensis* extract is an effective oral care product suitable for use as an effective dental caries prevention agent.

## 1. Introduction

Dental caries comprise one of the oral diseases that have inflicted mankind for a long time, gradually becoming a cultural disease with the prevalent use of sugar as a sweetener [1,2]. Such a form of dental caries is an infectious disease caused by microorganisms, and is a multi-factorial disease that leads to the destruction and loss of the dental ridge due to the interaction of bacteria, food, and saliva in the dental plaque [3,4,5,6]. It is said that 80–90% of Koreans suffer from dental caries at least once in their lifetime, and 60% of adults worldwide contract dental caries [7].

Plaque, the main cause of dental caries, is formed when a film is formed on the tooth surface due to glycoproteins derived from saliva and the oral microorganisms that adhere to it [8]. The bacteria involved in the initial adhesion are aerobic, gram-positive, genus *Streptococcus*, genus *Actinomyces*, and so forth [9]. *Streptococcus mutans* (*S. mutans*) is a main type of bacteria that causes dental caries, secretes glucosyltransferase (GTFase) and catalyzes the synthesis of adhesive, water-insoluble glucans using sucrose as a substrate [10]. This adhesive, water-insoluble glucan attaches *S. mutans* to the tooth surface, enabling adhesion and proliferation of other microorganisms in the plaque formation [11]. In addition, after attaching to the tooth surface, *S. mutans* produces acid by fermenting lactic acid from polysaccharides ingested from food [12]. When the pH of these organic acids drops below 5.5, the hydroxyapatite crystals in the enamel are decomposed or demineralized, causing dental caries [13]. Therefore, in order to prevent dental caries, it is important to effectively remove the biofilms of the genus *Streptococcus* and genus *Actinomyces*, which are involved in the formation of early plaque. For this, antibiotics such as chlorhexidine, triclosan, and cetylpyridinium chloride (CPC) are effective [11]. However, since these mouthwashes contain chemically synthesized agents and some commercialized products have antibiotics added to them, their long-term use can cause adverse side effects, including antibiotic resistance [14].

Accordingly, there is an increasing demand for mouthwash made from natural products that have fewer side effects from chemical compounds and are safe for long-term use [15,16,17,18]. It has been reported that *Asarum sieboldii*, *Syzygium aromaticum, Machilus hunbergia, Glycyrrhiza uralensis, Mentha canadensis, Platycodon root, Cnidium officinale,* and ginkgo leaves have high antibacterial effects as natural mouthwash agents against oral disease-causing bacteria [19]. Various medicinal plants, such as *Morus alba, Reynoutria japonica*, cinnamon, Star anise, *Chrysanthemum indicum*, and Barbary fig have been reported to inhibit the growth of *S. mutans* [1,20,21]. In addition to these substances, bloodroot, sage, myrrh, licorice root, *quercus infectoria gal, vespae nidus, Cratoxylum formosum*, chicory, and *Prunella vulgaris* have been reported to contain substances that can prevent dental caries [22].

*Glycyrrhizae Radix, Licorice, and Glycrrhiza* have the following scientific names: *Glycyrrhizae Uralensis Fisch Et, DC* are perennial herbs belonging to the Leguminosae family; *Glycyrrhizae uralensis Fisch., Glycyrrhizae inlata Bat., Glycyrrhizae glabra L*.; and their roots and stems have been globally used as a natural sweetener and medicine for a long time [23]. The pharmacological action of *Glycyrrhiza uralensis* includes the restoration of liver and intestine function as it binds to toxic substances in the liver and intestine. It subsequently detoxifies and acts on drug addiction, infection, urticaria, dermatitis, and eczema, and also provides anti-tussive action, anti-histamine action, and anti-acetylcholine action [24]. Alleviation of pain caused by rapid muscle or tissue tension, weight gain, white blood cell increase, diuretic action, anti-inflammatory action, anti-cancer action, detoxification action, anti-oxidant action, circulatory system action, anti-microbial action, and so forth have also been reported [25]. Since *Glycyrrhizae uralensis* has sweet-tasting properties, it is commercially used as a sweetener for chewing gum, chocolate candy, tobacco, smoking mixture, chewing tobacco, and snuff in some parts of Europe and Asia, and is often used as a masking agent for bitter drugs, such as aloe and quinine [26]. In addition, one of the active ingredients of *Glycyrrhiza uralensis*, saponin, has a surfactant property and is used to promote absorption of poorly absorbed drugs, such as anthraquinone glycosides, including emonin and optusin [27,28]. As such, *Glycyrrhiza uralensis* has the effect of regulating and supplementing the properties of other drugs and reducing toxic intermediates, so it is widely used in oriental medicine prescriptions to increase the activity of herbal medicines [29]. While it is composed of 14 compounds, it has been reported that mainly, six of the following components are contained: glycyrrhizin, liquiritin, liquiritin apioside, isoliquiritin, isoliquiritin apioside, and liquiritingenin [30]. It has been reported that the substances contained in the *Glycyrrhiza uralensis* extract can effectively inhibit *S. mutans*, which is the main causative agent of dental caries [31].

*Glycyrrhiza uralensis* extract with concentrations of 50 and 100 μg/mL showed a cell viability of 70% or more. The *Glycyrrhiza uralensis* extract exhibited excellent antimicrobial effects against *S. mutans*. Thus, the *Glycyrrhiza uralensis* extract at a concentration of 100 μg/mL was considered a biocompatible, naturally derived extract [32]. Specifically, licoricidin, licorisoflavan A, licorisoflavan C, licorisoflavan D, licorisoflavan E, and Glycyrrhizol A have been reported to inhibit effectively [33]. Although there are many in vitro studies on *Glycyrrhiza uralensis* and the bacteria that cause dental caries, there are very few studies that investigate the changes in the oral environment to confirm its clinical efficacy. Marchetti et al. [34] reported that the oral rinse was effective in inhibiting plaque regeneration within 3 days of application of an alcohol-containing essential oil. Thus, this study confirmed the changes in the oral environment for 5 days after using the mouthwash.

This study was conducted based on the hypothesis that the use of mouthwash containing *Glycyrrhiza uralensis* extract would have an anti-dental caries effect. Therefore, by analyzing the effect of using mouthwash containing *Glycyrrhiza uralensis* extract on the bacteria that cause dental caries and acid production degrees, this study aims to provide basic data on the efficacy of oral gargle solutions for dental caries prevention.

## 2. Materials and Methods

### 2.1. Study Participants

**Figure 1:**
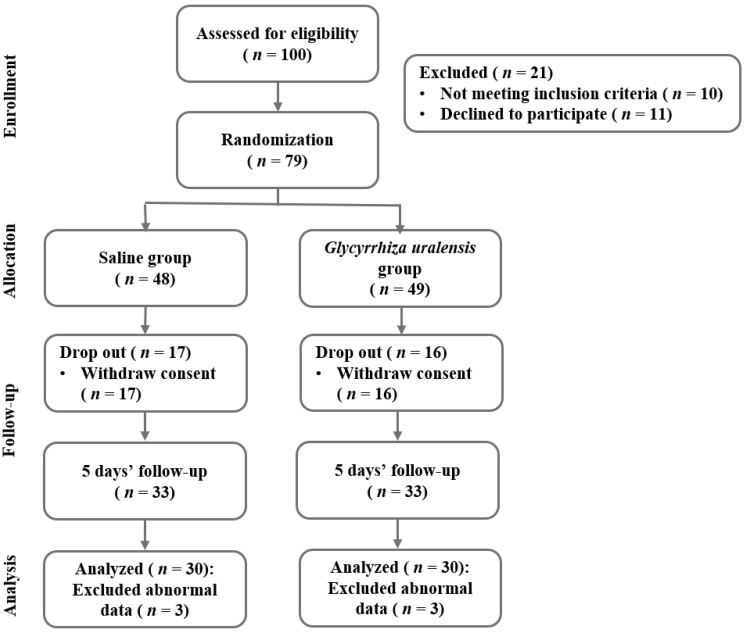
Research flow diagram.

Sample size was determined using the G* Power 3.1 program (Heinrich-Heine-University, Düsseldorf, Germany). The number of participants needed for the independent *t*-test with a significance level a = 0.05 bilateral test, power = 0.8, and effect size = 0.7 was 68. The initial sample size was planned at 96 considering a dropout rate of 40%, and 100 participants were actually included in this study. The dropout rate was set at high since the subjects were college students or working adults. A total of 100 subjects were screened and 79 were randomly assigned to the saline gargle group or *Glysyrrhiza uralensis* extract gargle group, after excluding 21 subjects who did not meet the inclusion criteria or refused to participate during the five-day study period. A total of 19 additional subjects were excluded from the five-day intervention phase, and data were finally analyzed for 60 subjects (Figure 1).

This study was conducted in accordance with the International Council for Harmonization of Technical Requirements for Pharmaceuticals for Human Use (ICH) guideline. The study was approved by the Kangwon National University (KNU) Institutional Review Board (KWNUIRB-2020-07-008-001, Chuncheon, South Korea). The purpose and procedure of the study were explained to all participants. Participants were also informed that refusal to participate would not disadvantage them in any way and they were free to withdraw from the study at any time.

### 2.2. Extraction of Plant Material

For this experiment, *Glycyrrhiza uralensis* grown in China was purchased from Cheongmyeong Co., Ltd. After adding 80% ethanol to 100 g of crushed *Glycyrrhiza uralensis*, the extraction was done in a heating mantle at 60 °C for 12 h. *Glycyrrhiza uralensis* extract was concentrated and lyophilized using a rotary vacuum evaporator (N-1300E.V.S. EYELA Co., Tokyo Rikakikai Co., Ltd., Tokyo, Japan) after filtration using filter paper (Advantec No. 2, Tokyo, Japan). The concentrated *Glycyrrhiza uralensis* was lyophilized using a freeze dryer (Ilshin Lab Co., South Korea) to obtain the *Glycyrrhiza uralensis* powder. The final concentration was used as a mouthwash containing 1 mg/mL *Glycyrrhiza uralensis* extract.

### 2.3. Study Design and Treatments

A randomized, double-blind, placebo-controlled trial was conducted on 60 patients who agreed to complete the questionnaire and met the inclusion criteria. A dental hygienist with more than 10 years of experience directly explained the objective of the study to the patients who visited the M dental clinic in Busan from October 2020 to June 2021. Among the patients with 16 or more remaining teeth, the subjects were selected by excluding those with severe dental disease (e.g., periodontitis, dental caries, dry mouth) (patients with enamel caries among dental caries were eligible to participate in the study, with the exception of patients with more than one dentine caries), those who smoke, those who have been diagnosed with sinusitis and/or rhinitis, those who are taking antibiotics, those with tongue problems (e.g., tongue cancer, glossitis), and those who received scaling within 2 months.

During the study, the same type of toothbrush and toothpaste was provided to all subjects, who were then instructed to use it during the study period. The subjects, who were given labeled gargles so that they could not identify whether they are in a study group or a control group, were instructed to gargle before going to sleep. Water or food ingestion was not allowed after gargling. A visit to the dentist for a clinical examination was conducted without any oral hygiene procedure, such as brushing and gargling in the morning. Data were collected in terms of gargle times, such as the baseline before gargle, immediately after gargle (Treatment), and 5 days after gargle (After 5 days) by two dental hygienists who were trained under the supervision of a dentist to increase the reliability of the investigator.

### 2.4. Clinical Examination

In order to ensure homogeneity of the oral environment, the subjects visited the M dental clinic in Busan one week before this study commenced, received oral examination, and were given oral scaling in order to establish an identical oral environment. A one-week recovery period was given after scaling to regenerate the gums. One week after the scaling was set as the baseline, the maxillary right first molar (#16), maxillary left central incisor (#21), mandibular left first molar (#36), and mandibular right central incisor (#41) were selected as the target teeth. The study group received 15 mL of *Glycyrrhiza uralensis* mouthwash while the control group received 15 mL of saline. This was performed for a total of 5 days. Cariview test and microbiological analysis were conducted as clinical indicators to confirm the inhibitory and preventive effects of dental caries in the oral environment.

### 2.5. Cariview Test

**Figure 2:**
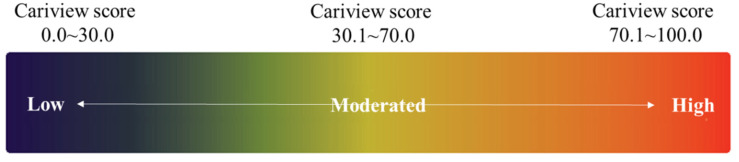
The criteria according to the pH value by the Cariview test.

Cariview™ kit (AIOBIO, Seoul, Korea) was used according to the manufacturer’s instructions in order to evaluate the acid production capacity of the dental plaque. The buccal surfaces of subjects #16 and #36 were thoroughly rubbed with a sterile cotton swab, and then immediately put into the culture solution. After culturing for 48 h at 37 °C in an incubator, 10 drops of the indicator in the kit were added, and color change was observed. A score was given based on the image taken using an optical analyzer (Allinone Bio, Seoul, Korea) according to the standards instructed by the manufacturer. The Cariview score means that the closer to 0, the higher the pH value, and the closer to 100, the lower the pH value. According to the criteria, subjects with a Cariview score of 0.0–40.0 are low-risk; 41.0–70.0, medium-risk; and 71.0–100.0, high-risk (Figure 2).

### 2.6. Microbiological Analysist

A total of #15 paper points were inserted to the gingival sulcus of four sites of two maxillary teeth (anterior and posterior) and two mandibular teeth (anterior and posterior) of the subjects for 10 s. Collected paper points were placed in a sterilized tube and were immediately stored at −20 °C until just before DNA analysis was extracted from the collected #15 paper points using the AccuPrep Universal RNA Extraction Kit (Bioneer, Daejeon, Korea). Extraction was performed according to the manufacturer’s instructions. OligoMix (YD Global Life Science Co., Ltd., Seongnam, Korea) and three oligonucleotides (Table 1) that react specifically to each bacterium were used [35]. A total of 9 μL of OligoMix, 10 μL of 2x probe qPCR mix (Takara Bio Inc., Shiga, Japan), and 1 μL of template DNA were combined to prepare the polymerase chain reaction (PCR) reaction sample. A 96-well plate with the PCR reaction sample was placed in the CFX96 Touch Real-Time PCR Detection System (Bio-Rad, Hercules, USA) to amplify the DNA. PCR conditions were as follows: initial denaturation at 95 °C for 30 s, denaturation at 95 °C for 10 s, and annealing for 30 s at 62 °C with 40 repeated cycles. The Bio-Rad CFX Manager Software program was used to calculate the cycle threshold (Ct) value, while the number of copies was derived by plotting the Ct value in the standard curve of each bacterium.

### 2.7. Statistical Analysis

IBM SPSS Statistics 24.0 from SPSS Inc., Chicago, IL, USA was used to evaluate significant differences. An independent *t*-test and chi-square test were performed to compare the demographic characteristics of the saline gargle group and the *Glycyrrhiza uralensis* extract gargle group. One-way ANOVA was used for time analysis based on gargle application, while Tukey’s test was performed as a post hoc analysis. To confirm normality in this study, skewness was 0.208 ± 0.184 and Kurtosis was −1.128 ± 0.365, so normality was satisfied. A significance level of *p* = 0.05 was applied in order to verify the significance between the groups.

## 3. Results

### 3.1. Study Population

There were more women than men in both the saline gargle group and the *Glycyrrhiza uralensis* extract gargle group. The mean age of the subjects in the saline gargle group was 44.27 years, while the mean age of the subjects in the *Glycyrrhiza uralensis* extract gargle group was 45.53 years. In both groups, there were more people without systemic disease, and there were more married subjects than single ones. There was no significant difference in the demographic variables between the two groups (Table 2).

### 3.2. Evaluation of Cariview Activity

The Cariview score was used to evaluate dental caries activity by measuring the acid production rate of the oral bacteria. For the dental caries activity test, there was no significant difference in the baseline between the saline gargle group and *Glycyrrhiza uralensis* extract gargle group (*p* > 0.05). For the Cariview scores, there were significant differences between the two groups when measured immediately after gargle application (Treatment) and five days after gargle application (After 5 days) (*p* < 0.05). There was no significant difference in temporal change based on the gargle application time for the saline gargle group, but it was significantly decreased for the *Glycyrrhiza uralensis* extract gargle group immediately after gargle application (Treatment) and at five days after gargle application (After 5 days), compared to the Baseline.

Results of investigating the risk of dental caries activity showed there was no significant difference between the saline gargle group and *Glycyrrhiza uralensis* extract gargle group at the baseline before gargle application (Baseline) and immediately after the gargle application (Treatment) (*p* > 0.05). There was a significant difference between the groups at five days after gargle application (*p* < 0.05). In terms of temporal changes, while there was no significant difference in the saline gargle group (*p* < 0.05), there were significant differences in the *Glycyrrhiza uralensis* extract gargle group from immediately after gargle application (Treatment) to 5 days after gargle application (After 5 days) compared to the Baseline (*p* < 0.05) (Table 3). In terms of the risk distribution, high risk was increased at five days after gargle application for the saline gargle group, and low risk was increased at five days after gargle application for the *Glycyrrhiza uralensis* extract gargle group, in which high risk was not found (Table 4). Based on the analysis of dental caries activity according to gender, there was no significant difference in the baseline regardless of the gender (*p* > 0.05), but there were significant differences immediately after gargle application (Treatment) and five days after gargle application (After 5 days) regardless of gender (*p* < 0.05). The *Glycyrrhiza uralensis* extract gargle group had lower significance than the saline gargle group (Table 5).

### 3.3. Dental Caries-Causing Bacteria in Subgingival Plaque

Three types of bacteria, *S. mutans*, *S. sobrinus*, and *Actinomyces viscosus (A. viscosus)*, were observed in both groups. When comparing the two groups for *S. mutans*, the saline gargle group and the *Glycyrrhiza uralensis* extract gargle group showed a significant difference at five days after gargle application for both the Maxilla and Mandible (*p* < 0.05). *S. sobrinus* showed a significant difference between the two groups only in the mandible immediately after gargle application (Treatment) (*p* < 0.05), while *A. viscosus* showed a significant difference between the two groups in the mandible from Treatment to After 5 days (*p* < 0.05).

In terms of change according to the application period, there was a significant difference between Treatment and After 5 days in the mandible for *S. mutans* (*p* < 0.05) in the case of the saline gargle group. In the case of the *Glycyrrhiza uralensis* extract gargle group, there were significant differences at After 5 days in the maxilla, and at Treatment and After 5 days in the mandible (*p* < 0.05). *S. sobrinus* showed a significant difference only at Treatment in mandible, while *A. viscosus* showed a significant difference at Treatment and After 5 days in mandible (*p* < 0.05) (Table 6).

## 4. Discussion

Oral health is part of overall health and is an essential element of physical health in terms of digestion and nutrition. Oral hygiene is also an important variable as a health index [36]. However, although the incidence of dental caries is increasing every year due to changes in dietary habits, a clear preventive and therapeutic agent has yet to be developed, resulting in many patients spending considerable medical expenses on dental care that are not reimbursed by medical benefits [37].

The causative bacteria of dental caries include *S. mutans, S. sobrinus, S. sanguinis, S. salivarius*, and *A. viscosus*. Studies on natural antibiotics that can replace chemical antibiotics like chlorhexidine, which have been used to treat oral diseases by inhibiting the growth of such oral bacteria, are being actively conducted to reduce various side effects [14,15,16,17,18]. Therefore, this randomized, double-blind, placebo-controlled trial confirmed the clinical effect of oral caries prevention and antibacterial action by applying mouthwash containing *Glycyrrhiza uralensis* extract. The Cariview test evaluates the dental caries activity by observing color changes using a colorimetric method that serves as an indicator for the pH of organic acids produced through overall bacterial culture [38]. This study confirmed that dental caries activity was decreased when *Glycyrrhiza uralensis* extract gargle was applied, and was gradually decreased as time passed. The risk of dental caries, obtained by dividing the dental caries activity score into three indexes, was also reduced. Upon comparison of differences by gender, the risk of dental caries activity was clearly reduced when mouthwash containing *Glycyrrhiza uralensis* was used regardless of gender. The results of this study confirmed that gargling with the mouthwash containing *Glycyrrhiza uralensis* extract for 5 days could simultaneously reduce the risk of dental caries and prevent dental caries.

Bacteria live in places that suit their physical characteristics, such as on the teeth, palate, gingiva, tongue, and buccal mucosa [39]. Oral pathogens can cause pain, eating disorders, and teeth loss by causing diseases such as dental caries and periodontal disease, and may also interfere with social life by acting as the main cause of bad breath [40]. Among them, *S. mutans*, a representative causative agent of dental caries, was not detected in the oral cavity after using mouthwash containing *Glycyrrhiza uralensis* for 5 days, while *S. sobrinus* had a significant difference only in the mandible immediately after the application of gargle. Since *S. sobrinus* has excellent acid production ability, it is known to increase the incidence of dental caries when coexisting with *S. mutans* [41]. In addition, since *S. sobrinus* is related not only to oral diseases, but also to systemic diseases, such as acute endocarditis, hemorrhagic stroke, pneumonia, temporomandibular arthritis, sepsis, ulcerative colitis, otitis media, and meningitis, continuous efforts to lower its levels in the oral cavity are needed [42]. In this study, there was a significant effect on *S. sobrinus* only in the mandible immediately after the application of the *Glycyrrhiza uralensis* gargle, and this is because the gargle remains longer in the mandible than the maxilla due to the nature of the gargle solution. Therefore, additional studies are needed so that the antibacterial action can be effectively exhibited throughout the oral cavity by using pine tree nots, ginseng, goldthread, mint, and *Spirodela polyrhiza* [43], which are known to provide excellent antibacterial action on *S. sobrinus.* By forming tartar, *A. viscosus* is known to be associated with oral diseases, such as root caries and periodontitis, and systemic diseases, such as sepsis, endocarditis, actinomycosis, and actinomycosis [44]. It was confirmed that the detection of *A. viscosus* in the mandible decreased immediately after using mouthwash containing *Glycyrrhiza uralensis* and at 5 days after use. These results are consistent with the in vitro studies that have already confirmed the antibacterial effect of *Glycyrrhiza uralensis* on the bacteria that cause dental caries [32,33,34].

Although oral mouthwash with natural agents is being studied, one of the reasons why it is not widely adapted is because most natural agents have a strong, characteristically bitter taste, so the overall preference is very low. Since *Glycyrrhiza uralensis* has a weak bitter taste, it is thought to be highly useful as a mouthwash ingredient. Based on the above results, the mouthwash containing *Glycyrrhiza uralensis* extract demonstrated a clear effect in reducing risk of dental caries activity due to decreased acid production ability. It also exhibited antibacterial effects due to significant suppression of the bacteria that cause dental caries. With this, the preventive effect on dental caries through clinical studies was confirmed. Mouthwash containing *Glycyrrhiza uralensis* extract is thought to play a role as a natural mouthwash with an excellent antibacterial effect for dental caries in the oral cavity, and its effectiveness as a natural antibiotic will contribute to the improvement of oral health by replacing the chemical-based mouthwash. The limitation of this study is that the long-term effect could not be confirmed due to the short study period. It was also not compared with various mouthwashes on the market. As a follow-up study, we plan to conduct comparative studies with commercially available mouthwashes, as well as conduct research on the development of mouthwashes that can show excellent effects at optimal concentrations by applying *Glycyrrhiza uralensis* extract at various concentrations.

## 5. Conclusions

The use of mouthwash containing *Glycyrrhiza uralensis* extract showed a decrease in the caries activity test. In addition, *S. mutans*, a representative type of bacteria that causes dental caries, was not observed in the oral cavity at 5 days after gargling. Therefore, the oral gargle containing *Glycyrrhiza uralensis* extract, which is a natural agent, will make a huge contribution in the improvement of oral diseases through its use and development as a preventive agent for dental caries. The extract has excellent antibacterial activity, is safe for the human body, and has few side effects because it does not contain any chemical compounds.

## Figures and Tables

**Table 1:** Primers and probes used in the real-time PCR assays.

Bacteria	Target Genes	Primers/Probe Sets	Amplicon Size (bp)
*Streptococcus mutans*	mannitol-specific enzyme II (mtlA) gene	5′-CAGCGCATTCAACACAAGCA-3′ 103 5′-TGTCCCATCGTTGCTGAACC-3′ 5′-HEX-TGCGGTCGTTTTTGCTCATGG-BHQ1–3′	103
*Streptococcus sobrinus*	16S ribosomal RNA gene	5′-GTACAACGAGTCGCAAGCCG-3′ 149 5′-TACAAGGCCCGGGAACGTAT-3′ 5′-FAM-TAATCGCGGATCAGCACGCC-BHQ1–3′	149
*Actinomyces viscosus*	sialidase (nanH) gene	5′-GCTCCCTCATGCTCAACTCG-3′ 5′-GATGATCTGGGCGTTGTCCA-3′ 5′-Texas Red-GAGCCGGTCCCCGACAAGAA-BHQ2–3′	140

**Table 2:** Characteristics of the subjects in the two groups.

Characteristics		N (%)		
		Saline	*Glycyrrhiza uralensis*	*p*-Value
* Gender	Male	7 (23.3)	9 (30.0)	0.771
	Female	23 (76.7)	21 (70.0)	
^¥^ Age (mean ± SD)		44.27 ± 11.5	45.53 ± 11.22	0.821
* Systemic disease	No disease	24 (80.0)	26 (86.7)	0.731
	Have a disease	6 (20.0)	4 (13.3)	
* Marriage	Single	14 (46.7)	13 (43.3)	1.000
	Married	16 (53.3)	17 (56.7)	

^¥^*p*-values are determined by the independent *t*-test (*p* < 0.05), * *p*-values are determined by the chi-square test (*p* < 0.05).

**Table 3:** Changes in the Cariview scores of the two groups.

Variables	Group	Mean ± SD			* *p*-Value
		Baseline	Treatment	After 5 Days	
Cariview scores	Saline	54.94 ± 6.95 ^a^	54779 ± 4.01 ^a^	60.22 ± 2.73 ^a^	0.056
	*Glycyrrhiza uralensis*	54.89 ± 7.55 ^a^	44.04 ± 4.13 ^b^	45.54 ± 4.91 ^b^	**0.001**
	^¥^*p*-Value	0.990	**<0.001**	**<0.001**	
Risk	Saline	2.07 ± 0.13 ^a^	2.00 ± 0.00 ^a^	2.17 ± 0.19 ^a^	0.057
	*Glycyrrhiza uralensis*	2.10 ± 0.15 ^a^	1.90 ± 0.15 ^b^	1.90 ± 0.15 ^b^	**0.017**
	^¥^*p*-Value	0.705	0.083	**0.004**	

^¥^*p*-values are determined by independent *t*-test (*p* < 0.05), * *p*-values are determined by one-way ANOVA and Tukey tests (*p* < 0.05). Different letters (a and b) by the presented statistically significant result of the post hoc Tukey tests. Values are means ± standard deviations; significant (bold); Risk code means 1 is low-risk; 2 is medium-risk; and 3 are high-risk.

**Table 4:** Distribution of the subjects according to their risk differences (*n* = 60).

Group	Risk	N (%)		* *p*-Value
		Baseline	After 5 Days	
Saline	Low	0 (00.0)	0 (00.0)	0.425
	Moderate	26 (86.6)	25 (83.3)	
	High	4 (13.4)	5 (16.7)	
*Glycyrrhiza uralensis*	Low	0 (00.0)	3 (10.0)	0.051
	Moderate	27 (90.0)	27 (90.0)	
	High	3 (10.0)	0 (00.0)	

* p-values are determined by Fisher’s exact test (*p* < 0.05).

**Table 5:** Comparison of changes in the cariview according to gender (*n* = 60).

Cariview	Female (*n* = 48)		Male (*n* = 12)	
	Saline (*n* = 24)	*Glycyrrhiza uralensis* (*n* = 24)	Saline (*n* = 6)	*Glycyrrhiza uralensis* (*n* = 6)
Baseline	51.19 ± 11.06	50.11 ± 11.25	71.46 ± 13.91	74.00 ± 13.91
*p*-value ^†^	0.746		0.770	
Treatment	54.15 ± 8.86	44.43 ± 7.94	57.35 ± 1.37	42.50 ± 10.07
*p*-value ^†^	**<0.001**		**0.005**	
After 5 days	60.97 ± 5.89	44.58 ± 10.28	57.35 ± 1.37	49.40 ± 7.12
*p*-value ^†^	**<0.001**		**0.023**	

^†^*p*-values are determined by independent *t*-test (*p* < 0.05); Values are means ± standard deviations; significant (bold).

**Table 6:** Clinical outcomes were observed between the groups.

Variables		Group	Mean ± SD			
			Before	Treatment	After 5 Days	* *p*-Value
*Streptococcus mutans*	Maxilla	Saline	41.81 ± 34.84 ^a^	45.30 ± 31.70 ^a^	35.71 ± 37.00 ^a^	0.868
		*Glycyrrhiza uralensis*	64.80 ± 51.50 ^a^	40.58 ± 21.40 ^a^	0.00 ± 0.00 ^b^	**0.002**
		^¥^*p*-Value	0.334	0.756	**0.017**	
	Mandibular	Saline	83.00 ± 57.91 ^a^	14.50 ± 11.35 ^b^	16.67 ± 14.30 ^b^	**0.000**
		*Glycyrrhiza uralensis*	83.00 ± 57.91 ^a^	10.50 ± 9.79 ^b^	0.00 ± 0.00 ^b^	**0.000**
		^¥^*p*-Value	1.000	0.490	**0.003**	
*Streptococcus sobrinus*	Maxilla	Saline	127,537.76 ± 98,642.17 ^a^	119,800.90 ± 110,133.95 ^a^	124,878.57 ± 71,101.00 ^a^	0.988
		*Glycyrrhiza uralensis*	153,728.50 ± 133,372.23 ^a^	42,408.68 ± 26,692.10 ^a^	110,578.89 ± 55,820.33 ^a^	0.059
		^¥^*p*-Value	0.688	0.071	0.677	
	Mandibular	Saline	56,727.96 ± 30,115.95 ^a^	56,101.40 ± 26,700.44 ^a^	56,808.07 ± 23,433.67 ^a^	0.998
		*Glycyrrhiza uralensis*	90,897.70 ± 65,432.98 ^a^	25,941.37 ± 13,166.15 ^b^	89,438.63 ± 56,597.73 ^a^	**0.032**
		^¥^*p*-Value	0.205	**0.009**	0.196	
*Actinomyces viscosus*	Maxilla	Saline	387528.97 ± 156553.26 ^a^	378413.10 ± 203472.95 ^a^	370,530.17 ± 149,524.05 ^a^	0.982
		*Glycyrrhiza uralensis*	279,862.30 ± 152,068.99 ^a^	253,726.33 ± 140,596.78 ^a^	245,293.78 ± 127,744.71 ^a^	0.888
		^¥^*p*-Value	0.182	0.173	0.097	
	Mandibular	Saline	274,498.13 ± 119,897.44 ^a^	281,772.50 ± 132,463.89 ^a^	281,772.50 ± 132,463.89 ^a^	0.992
		*Glycyrrhiza uralensis*	210,164.80 ± 134,786.75 ^a^	55,076.30 ± 20,612.73 ^b^	32,903.89 ± 10,128.22 ^b^	**0.000**
		^¥^*p*-Value	0.333	**0.000**	**0.000**	

^¥^*p*-values are determined by independent *t*-test, * *p*-values are determined by one-way ANOVA and Tukey tests (*p* < 0.05).; Different letters (a and b) by the presented statistically significant result of the post hoc Tukey tests; Values are means ± standard deviations.; significant (bold).

## Data Availability

The data presented in this study are available on request from the corresponding author.
